# “No More Insecurities”: New Alternative Masculinities' Communicative Acts Generate Desire and Equality to Obliterate Offensive Sexual Statements

**DOI:** 10.3389/fpsyg.2021.674186

**Published:** 2021-05-25

**Authors:** Harkaitz Zubiri-Esnaola, Nerea Gutiérrez-Fernández, Mengna Guo

**Affiliations:** ^1^Faculty of Education, Philosophy and Anthropology, University of the Basque Country, San Sebastian, Spain; ^2^Faculty of Psychology and Education, University of Deusto, Bilbao, Spain; ^3^Department of Sociology, University of Barcelona, Barcelona, Spain

**Keywords:** self-confidence, attraction patterns, offensive sexual statements, new alternative masculinities, desire, communicative acts

## Abstract

To justify attraction to Dominant Traditional Masculinities (DTM) and lack of attraction to non-aggressive men, some women defend opinions such as “there are no frigid women, only inexperienced men”. Such statements generate a large amount of sexual-affective insecurity in oppressed men and contribute to decoupling desire and ethics in sexual-affective relationships, which, in turn, reinforces a model of attraction to traditional masculinities that use coercion, thus perpetuating gender-based violence. New Alternative Masculinities (NAM) represent a type of masculinity that reacts to reverse such consequences with communicative acts, in which they state that women who support such discourses have never met a NAM man or have never experienced a successful sexual-affective relationship where passion, love, desire, and equality are all included. This article presents data analyzing these communicative acts (exclusory and transformative; language employed and consequences) to ultimately find the key to NAM communication that would contribute to changing attraction patterns. The data was collected using communicative daily life stories of three heterosexual white men and one heterosexual white woman, between the ages of 30 and 40. Findings emphasize the importance of self-confidence manifested by NAM men when communicating about sex and facing these offensive mottos in the presence of other men and women. Findings also demonstrate that supportive egalitarian relationships encourage the emergence of self-confidence in NAM men and that NAM men's self-confident communicative acts foster healthy relationships and obliterate coercive ones.

## Introduction

The scientific literature on masculinities and communication has disregarded the influence of men's self-confidence and self-esteem in shaping whether they are considered attractive or not. While there are communicative acts that can contribute to enhancing a man's attractiveness, others can diminish it. A key aspect of these interactions is how the man reacts, leading to an increase of his attractiveness or otherwise. Therefore, most studies that analyze men's self-confidence are psychology-centered and do not consider communicative acts as a key explanatory element (Long and Martinez, 1997; Levant and Pollack, 2008; Reilly et al., 2014). In this study, we include various contributions from gender studies and linguistics that have explored the influence of men's communication to shed light on this topic (Edley and Wetherell, 1997; Renold, 2001, 2004; Mac an Ghaill, 2003; Connell and Messerschmidt, 2005; Bogg and Ray, 2006; Chopra, 2006; Korobov and Thorne, 2006; Brown and Macdonald, 2008; Itakura, 2014; Reda and Hamdan, 2015).

Thus, psychological investigations have mostly studied men's emotional development and its connection with self-confidence (Long and Martinez, 1997; Levant and Pollack, 2008; Reilly et al., 2014). From gender studies, the analysis of men's communication has focused on the gendered discourses connected with hegemonic and non-hegemonic masculinities (Connell, 1994, 2012; Edley and Wetherell, 1997; Renold, 2001, 2004; Mac an Ghaill, 2003; Connell and Messerschmidt, 2005; Bogg and Ray, 2006; Chopra, 2006). Linguistics researchers have explored messages from the media, communicative acts and interactions that promote hegemonic and non-hegemonic masculinities (Edley and Wetherell, 1997; Korobov and Thorne, 2006; Hall et al., 2011; Portell and Pulido, 2012).

However, the role of non-hegemonic men's communicative acts that are rooted in self-confidence and change general attraction patterns among heterosexual women are not deeply analyzed, nor in the way in which offensive sexual statements influence non-hegemonic men's self-confidence. This article will present findings with relevant empirical data on the influence of these communicative acts. In this regard, we will start from a theoretical conceptualization based on the definition of the three types of masculinities (Díez-Palomar et al., 2014). These three types—which are widely explained in the introduction of this Special Issue—are New Alternative Masculinities (NAM, hereinafter), Oppressed Traditional Masculinities (OTM, hereinafter), and Dominant Traditional Masculinities (DTM, hereinafter). Alternatively, the theoretical perspective on communicative acts (Soler and Flecha, 2010) that are extensively described in the introduction will be the sociolinguistic approach that is used to analyze men's and women's communicative acts. This approach pays particular attention to the social consequences of both men's and women's communicative acts.

The article is divided into four sections. The first section provides a literature review and considers the aforementioned perspectives on the types of language uses and discourses connected with hegemonic masculinity (including DTM), non-hegemonic masculinities (including egalitarian men, new masculinities, OTM, and NAM within this typology) and self-confidence. The second section presents the description of the study carried out, including the theoretical and methodological approaches used for the data analysis. The third section shows the findings that are obtained through this analysis, presenting the consequences of utilizing exclusionary and transformative communicative acts, especially regarding sexual issues, in shaping men's self-confidence and insecurity. At the end, the conclusions with further research implications are presented.

## Language and Discourses on Non-hegemonic Masculinities and Self-Confidence

Different studies have tackled the relationship between non-hegemonic men's communicative acts and self-confidence from different points of view. In particular, research conducted in gender studies, psychology and linguistics has given greater attention to this topic. In the following section, a review of the evidence from these three disciplines will be presented and considered according to its connection with other complementary aspects of our analysis, such as men's insecurity, sexual statements, desire, and attraction.

Drawing on a gender studies perspective, Raewyn Connell (in Kessler et al., 1985) initiated men's studies with her work on “hegemonic masculinity” in schools. In that analysis she argued, following Gramsci, that boys and girls construct gender identities in school that become predominant. These identities are mostly shaped based on gendered discourses that reinforce male power and competitiveness. More recently, Connell (2012) affirmed that the globalization process is contributing to changing this situation and disseminating gendered discourses that foster non-hegemonic masculinities, such as the egalitarian men's movement. However, these non-hegemonic masculinities are not always socially valued; in fact, there are several studies that illustrate the types of discourses and language uses that generate insecurity for men exhibiting these masculinity types (Edley and Wetherell, 1997; Renold, 2001, 2004; Mac an Ghaill, 2003; Bogg and Ray, 2006; Chopra, 2006). For instance, in the school context, these studies identify bullying practices against non-hegemonic boys (Renold, 2001, 2004; Mac an Ghaill, 2003; Lawson, 2013). Renold (2004) calls them “other boys,” showing the typology of the language and words that are commonly used by peers to bully non-hegemonic males. Words such as “swots” and “geeks” undermine these boys' level of attractiveness (Renold, 2001, p. 373). Duque and Teixido (2016) analyzed bullying related to homophobia, biphobia, transphobia, and gender violence in the school context and identified several actions to prevent and overcome it, among which is breaking the silence by taking a stand against violence, and bystander intervention, which consists on protecting those who suffer violence, so that relationships based on solidarity are created.

The scornful language has extremely negative consequences in non-hegemonic boys as they consequently experience marginalization and damaged self-esteem. Furthermore, Renold (2001) also underlines that dominant girls repeatedly humiliate and bully these boys, generating a deteriorating atmosphere in the class and influencing these boys' social reputation. The damaged social reputation is persistent in different contexts where non-hegemonic men break with normative gender rules. For instance, Chopra (2006) discusses the role of male domestic workers in India and the harmful effects caused by the speech styles and body language that their employers use toward them. Despite male domestic workers using body language and speech acts that are connected with virtue and innocence, employers do not place value on these acts and usually utilize hierarchical forms of communication to harass them: “Often speech is literally replaced by a bell to summon a worker. The bell asserts hierarchy and initiates required actions literally without a word being spoken” (Chopra, 2006, p. 161).

In contrast to previous studies, Edley and Wetherell (1997) perform an analysis connected with gendering sports discourses. In this regard and based on a deep analysis of young, non-hegemonic men's conversations, they identify the existence of positive discourses on non-hegemonic men in which the consequences foster their social acknowledgment. These discourses are constructed when non-hegemonic men display self-confidence with their own masculinity that is widely accepted and valued by their surroundings. Although these men do not normally practice traditional masculine sports such as rugby and are not engaged in “macho” and chauvinist dynamics, their manhood is commonly questioned. In contrast, rugby players who maintain these chauvinist practices do not experience such questioning of their masculinity. In this way, in spite of the previously mentioned transformative elements, the findings corroborate the persistence of constructing hegemonic discourses that discredit non-hegemonic men as “sissies” and “poofs.” Piedra (2017) also analyzed a case in which boys between the ages of 8 and 19 who practiced rhythmic gymnastics, traditionally seen as a female sport, were insulted and teased for doing so.

Tsuda (2020) analyses the attractiveness of hegemonic and non-hegemonic masculinities and how they entangle with ethnic features. Men framed in non-hegemonic masculinities are considered to have a lower value in the field of sexual-affective relationships. Women state that these men “don't fit the stereotype of the sexy, desirable man.” Women depict them as “socially inept”: they “don't have a personality,” “they can't carry on a conversation and they are awkward” (Tsuda, 2020, p. 9–10). Some of these non-hegemonic men try to overcome stereotypes by performing the characteristics of hegemonic masculinity, which is defined as aggressive and domineering over women, but these acquired hegemonic characteristics do not raise the value of these non-hegemonic men. Nevertheless, Tsuda (2020) emphasizes that non-hegemonic egalitarian masculinities are beginning to emerge and begin to challenge hegemonic masculinity, focusing mainly on the ethical components of non-hegemonic masculinities.

Focusing on psychological analyses of non-hegemonic masculinities, as had been previously introduced, more attention is paid to the configuration of their self-confidence than the influence of language and communication. Firstly, it is relevant to highlight the work done by Reilly et al. (2014) as they study the relationship between two elements of men's behavior to comprehend certain personal changes: heterosexual men's conformity with masculine norms and self-confidence. The findings show that men's acceptance of masculine normativity is strongly connected with having strong self-confidence and self-esteem. In other words, when men are comfortable with a traditional gendered identity, they emotionally feel positive. Similarly, Hoffman et al. (2000) discovered a strong connection with the sex-role conception related to men's self-confidence. Thus, they found that men who scored high on gender self-acceptance are happier with themselves and their self-confidence is consequently more robust. In the same way—in a previous study developed by Long and Martinez (1997) on Hispanic professional men's self-confidence—self-esteem and self-acceptance became an important explanatory variable in understanding the levels of confidence that these men developed in their workplaces.

In contrast to the preceding studies in the field of psychology, Levant and Pollack (2008) introduced interactions in their psychological analysis of men's self-esteem and self-confidence. They explored the emotional changes that fatherhood implies for men who are deeply involved in the fatherhood role. The investigation performed by these authors suggests that fatherhood offers men the opportunity to transform their self-structure based on their emotional dedication to their children's well-being. In this sense, men's interactions with children contribute to transforming them into less selfish, more egalitarian people with higher self-esteem and self-confidence.

From the linguistic perspective, several studies have explored the incidence of media discourses and communicative acts for the social construction of masculinity. However, these analyses rarely include self-confidence as a key element in men's socialization processes but include the study of the consequences of these discourses and communicative acts both in men and women. Hall et al. (2011) have investigated media messages on “metrosexual” men. This typology was momentarily categorized as part of new masculinities simply because “metrosexual” men are seen as interested in fashion and personal care. Thus, they performed a discourse analysis of messages exchanged in online forums dedicated to “metrosexual” men's discussions. The findings coincide with previous contributions and underline the effects of these online debates on the reproduction of the hegemonic masculinity as a successful male model, especially the promotion of hypermasculinization (Harris and Clayton, 2007).

In their analysis of males and females' heterosexual appeal, Bogg and Ray (2006) found analogous results based on messages from youth-centered magazines regarding university students' sexual interests. In this regard, the authors surveyed men and women by showing them images of male and female models from this type of magazine. The findings illustrate the effects that result in women's perception of middle-age “non-macho” images, in which women employed words such as “dad” or “older gentleman” to define these men's images and used unappealing adjectives such as “nerdy,” “geeky,” “dorky,” and “too preppy” that deprive them of sexual appeal. These results also support previous findings on the social construction of desire and attraction that demonstrate how linking seriousness and ethics in men's descriptions eliminates any link with sexual desire (Flecha and Puigvert, 2010).

In the field of linguistics, a study developed by Portell and Pulido (2012) sheds light on the connection between non-hegemonic masculinities and self-confidence. The authors developed a qualitative study on the communicative acts that men and women perform in various daily settings, such as at schools or companies. They pay special attention to the communicative acts that promote egalitarian masculinities in these spaces. In this regard, they identify that these conversations and interactions, which are based on men's self-confidence, are crucial to becoming a respected man. The following quote from a young student at a vocational school that was captured by the authors perfectly exemplifies this reality: “He has to be someone who stands out, who claims your attention, not just a stereotype but (…) Yes, somebody who is self-confident and who inspires, therefore, confidence and security” (Portell and Pulido, 2012, p. 75).

Isaksen (2017) found that from a non-hegemonic masculinity approach, negative representations can be defied through the humorous, calm, and laid-back use of rhetoric that ridicules the attacks and shows that this non-hegemonic masculinity is that of a bold, confident and cool man who is able to neutralize the attacks that want to represent him as an outsider.

Gómez (2015) also shows the importance for egalitarian men of the attitude they adopt toward other people, with self-confidence being fundamental, in order to be attractive and thus overcome situations in which they are dominated by discourses that promote double standards, gender-based violence and resignation in the face of unjust situations. Likewise, Villarejo et al. (2020) demonstrated that audiovisual products such as movies and videogames only created attraction to non-violent egalitarian masculinities when these characters showed self-confidence in relating to others, implying that the ethical component was necessary but not enough to generate attractiveness toward NAM. The key challenge of NAM is to embody ethics and attractiveness in a social context in which peer communicative interactions influenced by the coercive dominant discourse (Racionero-Plaza et al., 2020) often empty egalitarian non-violent men of their attractiveness and make violent masculinities attractive (Racionero-Plaza et al., 2021). In sexual-affective relations, coercion can occur both in verbal and non-verbal communication, and, therefore, the solution is necessarily based on taking into account not only speech acts, which are essential, but also other types of communicative acts (Flecha et al., 2020). The coercive dominant discourse imposes the link between attraction and violence, influencing the socialization that occurs from birth, in settings such as school, family, circles of friends, and audiovisual products, and therefore prevention is necessary in all of these settings (Aiello et al., 2018; Rios-Gonzalez et al., 2018, 2019; Ruiz-Eugenio et al., 2020; Villarejo et al., 2020).

In this line of prevention, Díez-Palomar and Mara (2020) found that the creation in the school context of dialogic high quality spaces for academic learning, free of violence and disruptive attitudes makes it possible for NAM boys to become more popular because they foster the link between the discourse of ethics and the discourse of desire. Meanwhile, in these contexts the attitudes of DTM boys, who tend to oppose academic dynamics and have aggressive attitudes toward peers, are not socially valued. Likewise, Redondo-Sama (2016) demonstrated that in organizations that are governed by democratic principles and where leadership is dialogic, NAM men become more visible to the detriment of traditional masculinities.

Drawing on these emergent results that connect the construction of non-hegemonic masculinities with communicative acts, in this article, we will take these results into account but also examine two particular elements that have not yet been studied: the effects of offensive sexual statements on non-hegemonic men as well as the influence of communication of men who follow some premises of NAM theory in changing attraction patterns. As will be later explained, there is no empirical evidence on these two elements, so new information on men's communicative acts and their social consequences will be provided, filling a gap in this field.

## Materials and Methods

### Theoretical Perspective and Methodological Approach

The theoretical perspective on communicative acts (Soler and Flecha, 2010) has been used to analyze verbal and non-verbal communicative interactions. This conceptualization has its origins in the scientific discussion on speech acts initiated by Austin (1996), Searle (1969), and Habermas (1987) who contributed to theories on the social basis of people's communication.

In addition to this theoretical framework, the methodological approach that has been followed in our study is Communicative Methodology (CM, hereinafter). CM's main objective is the construction of useful knowledge for the achievement of social transformation, and fulfilling this objective requires taking most of the relevant scientific contributions on the social sciences into account (Gómez et al., 2010). Among these contributions are Chomsky's (1988) universal grammar, Mead's (1934) symbolic interactionism, Habermas' (1987) theory of communicative action and Beck's (1992) conception of reflexive modernity.

CM attempts to establish an intersubjective dialogue without interpretative hierarchies between the researchers and subjects (Gómez et al., 2019; Soler and Gómez, 2020). The former contribute relevant scientific knowledge of the investigation and the latter provide information on their daily lives on the topic that is discussed. Afterward, transformative and exclusionary dimensions guide the expected data analysis in which the results show, on the one hand, that these barriers create difficulties in people's life, and on the other hand, that these successful mechanisms help them overcome these barriers (Pulido et al., 2014).

### Selection of Participants

The equations should be inserted in editable format from the equation editor. The selection criteria of the sample included (a) men who have felt insecure in their sexual and affective relationships due to offensive sexual statements they have been told or who know men who have been told these statements; (b) men who have not felt insecure after hearing offensive sexual statements or know men who remained self-confident in the face of such statements; (c) men who have felt insecure when facing these offensive sexual statements but have recently become more self-confident and know men who have experienced a similar change; and (d) women who have performed these offensive sexual statements or know women in their immediate surroundings who have performed these statements.

The participants in this study were three heterosexual white men and one heterosexual white woman, between ~30 and 40 years old (Table 1). These four participants have different work profiles and come from diverse socio-economic backgrounds. More details about their social identities and sexual and affective relationships are described below.

**Table 1:** Participants' profile.

***N***	**Name**	**Age**	**Profile**
1	Alejandro	35 years old	University professor
2	Xavier	38 years old	Secondary school teacher
3	Teresa	34 years old	Educator in leisure time
4	Enric	36 years old	Employer in a telecommunication enterprise

#### Participant 1: Alejandro

Alejandro is a Chilean man who comes from a working-class family. In Chile, he underwent vocational training to become an electrician because it was a commonly sought occupation in his region. After his professional education, he managed to attend college. While working as an electrician, he met a Spanish girl who is currently his wife. They relocated to Spain and had two sons. In Spain, he earned a PhD and became involved in an egalitarian men's association. This involvement has helped him become more self-confident in terms of rejecting his friends' chauvinist attitudes. In addition, as Alejandro asserts, he feels as sexually attracted to his wife as the day they met.

#### Participant 2: Xavier

Xavier belongs to a middle-class family and attended a private school as a child. He completed a degree in sociology and is now working as a secondary school teacher. He has always defined himself as an egalitarian man who does not want to follow hegemonic masculine practices. During his adolescence, he felt pressured by his family and friends in this regard but he remained confident about his gender identity. When he was in his twenties, he began participating in an egalitarian men's association and commenced a fulfilling sexual-affective relationship that has made him more self-confident. He has been with the same woman for 14 years and they now have two sons.

#### Participant 3: Teresa

Teresa has always lived in a humble working-class neighborhood in a large city in northern Spain. She did not finish secondary education as she was assigned to a remedial educational program. After working as a volunteer for an NGO in her neighborhood, she began working as an educator in leisure time. Teresa had various sexual and affective relationships that did not satisfy her, but a decade ago she fell in love with an egalitarian-minded man who is her current husband. Teresa and her husband's childhood friends are very skeptical about their egalitarian and sexually satisfactory relationship, so they are often questioned in this regard.

#### Participant 4: Enric

Enric comes from a middle-class family and is currently living in a working-class neighborhood in a northern Spanish city. Enric did not finish secondary education, but at 35 years old he passed the university entrance exam. Presently, he is an employee of a large telecommunication company where he often faces scornful sexual comments from his boss. He considers himself an egalitarian man with a high level of self-confidence, and he feels fortunate because his last sexual-affective relationship is very successful in terms of attraction and equity.

All the names used for the analysis of subjects' communicative acts are pseudonyms.

### Data Collection

The data collection was conducted using a communicative daily life story. This technique aims to gather participants' reflections about their past, present and future to interpret their lives with researchers (Gómez et al., 2010). Therefore, in our study, participants and researchers share their quotidian and scientific knowledge on communicative acts that are linked with (1) sexual statements against OTM men, and (2) NAM men's self-confidence-based language. Due to the interviewee's life experiences, they easily recognized several situations where these communicative acts emerge. The conversation allowed the research team to gather information regarding not only the interviewee's direct personal experiences but also those of their close friends and family who had experienced the relevant communicative acts as revealed by the study participant. These indirect communicative acts (experienced through a third person) have greatly contributed to the richness of the data gathered as the communicative acts were not only reproduced but the posterior reflection between the persons involved and our interviewee was also collected. In fact, this prior understanding helped develop more profound reflections and construct a dialogic knowledge about the incidence of language uses in people's lives.

### Data Analysis

The study presented in the article analyses two main aspects of language use: (1) exclusionary communicative acts that generate sexual-affective insecurity in oppressed men; and (2) transformative communicative acts based on non-hegemonic men's self-confidence when they communicate about sex and attraction. In this regard, the results will present these two dimensions of communicative acts that emphasize their social consequences. The objective of the data analysis is not to carry out an exhaustive examination of the interviewees' biographies; on the contrary, the objective is to study significant communicative acts that the interviewed subjects disclose concerning the effects of sexual statements aimed at OTM and NAM men. These significant communicative acts provide information about scornful language uses but also provide new evidence on the typology of language that transforms this dynamic and its social consequences.

### Ethics Statement

The current study was reviewed and approved with the number 202102231 by the Community of Researchers on Excellence for All's (CREA) Ethics Committee, which considered that present research meets the criteria established in the European Comission (2013) and the European Union's Charter of Fundamental Rights (2000/C 364/01) (European Union, 2000).

### Limitations

In the data collection process, we found some limitations regarding the objective of collecting peoples' communicative acts. These limitations include four main elements: (1) assurance of participants' sincerity about third persons' conversations, (2) the inability to identically reproduce people's disclosed communicative acts, (3) memory difficulties regarding interviewees' previous conversations, and (4) the limited number of subjects interviewed.

## Findings

### Exclusionary Communicative Acts: Generating Sexual-Affective Insecurity in OTM Men

The qualitative data confirms the existence of communicative acts that negatively influence men's self-confidence, sexually speaking. These exclusionary communicative acts performed by women and men are particularly affecting men who, considering our data, respond to the OTM model, which renders their egalitarian attitude less relevant and less attractive. For instance, Alejandro's daily life narrative presents different situations in which these exclusionary communicative acts emerge. In this regard, he relates one of these situations involving a dinner that he and his wife have with Pedro, his best friend, and his wife, Lucía. Alejandro defines Pedro as a “good guy” who strives to avoid conflict with others; however, Lucía is radically different and she complains often about many issues. In this situation, Alejandro remembers that Lucía made several comments to Pedro that discredited him at a sexual level. In fact, she blamed Pedro for not getting her pregnant. She also attacked his manhood, saying he was not “masculine” enough, and that he is not good in sexual relationships. The following three sentences illustrate the offensive sexual statements by Lucía:

I am not pregnant because of him. He cannot impregnate me, he does not hit the mark, he has no force and he is not masculine.

It is his fault because he does not have enough sexual potency.

He is very bad in bed.

From Alejandro's perspective, Lucia's offensive comments made Pedro begin to feel insecure. This situation made him nervous and he did not know what to say in the face of his wife's provocations. Alejandro described how Pedro blushed because he felt self-conscious about his inabilities and he tried to switch to another topic. Later, when Alejandro and Pedro discussed the situation, the latter justified his wife's comments: “Lucia was feeling so nervous; she has a lot of work.”

Similarly to Alejandro's experiences, Xavier has witnessed several situations in which one of his best friends, Jordi, was targeted with offensive statements from his girlfriend, Susana. Xavier describes Jordi as a very romantic man who always showed passion and devotion to his girlfriends, but these sentiments are usually not reciprocated. In this case, Xavier heard two comments that Susana repeatedly said about Jordi. One of these comments referred to Jordi's respectful attitude that did not generate any desire in her. Thus, they reinforced Susanna's perception of him as only a friend. Indeed, she argued that this lack of excitement was due to Jordi's behavior because he was always doing what she wanted and treated her too well.

They had been together for 2 or 3 months. She described him as a friend because, basically, she did not feel sexually attracted to him. While she was with this guy, other guys called her every day. She hid this information and did not say anything to him. During this period, she said “He was very much behind me. He was too good of a person because he treated me very well.”

Susana's second comment illustrates how the use of offensive sexual statements about Jordi was a way to justify her lack of interest. Thus, she openly remarked that he was not bold enough and never took the initiative in their sexual relationships:

At the end of the relationship, she made offensive sexual statements, like “I wanted to make love but he did not because he was not bold enough,” or other comments like “He needed more bravery to make love.” That is, more self-confidence.

Xavier also detailed the negative consequences of these comments on Jordi. The consequences are predominantly linked to a significant disaffection and insecurity for sexual and affective relationships. Jordi suffered greatly observing how his dedication increases his girlfriend's apathy. After that experience, he did not want to initiate any relationships or sexual affairs: “This experience negatively impacted him a lot—I guess, also for his initial motivation and then for being totally in love and it not being returned—to the point of not wanting any relationship or partner, or any girl with whom make love.”

In her narrative, Teresa also shared several examples of how her female friends used to make exclusionary communicative acts that some women often do to ridicule men they define as “wimps.” She narrates some discussions she had with one of her friends, Maria, about her sexual-affective relationships. Teresa likes to share experiences of intimacy with her friends but recently has been feeling very tired of listening to how they, including Maria, are always talking unenthusiastically about their partners, using negative adjectives such as “dumb” or “pain in the neck.” In this regard, Teresa explains that sometimes these conversations include offensive sexual statements that undermine men's attractiveness. In the next quote, Teresa recalls one of the comments that Maria made about the lack of sexual excitement she has with her husband, insisting that she prefers a man who treats her badly because otherwise she loses interest.

He is very understanding and so quiet, and this makes me feel that he is a wimp, that he is bad in bed, and this type of man doesn't excite me at all. I prefer to care than to be cared for, a man should comply and hit me, and if not, I don't give a damn about him.

As a result of these frequent comments and scornful statements, Maria and her husband had a dull sexual and affective relationship. Analogous situations were experienced by Teresa's friends; their partners' self-esteem is affected because they feel distance from their wives and girlfriends. In contrast, Teresa sees that her friends frequently question her relationship because she is sexually excited for her husband, and they sometimes ask her: “Shit! And he never gets angry? He is never aggressive? Are you sure that you are ok with him?”

Finally, more data on exclusionary communicative acts are found in Enric's daily life narrative, in which he introduces some examples of men's perceptions of these sexually offensive statements that women sometimes made. He underlines that he considers his male co-workers very respectful and assertive with their partners and female co-workers. However, in their conversations about sexual and affective relationships, they feel insecure because they perceive that their girlfriends are the ones who make the decisions in this regard. This situation contributes to labeling them as “wimps,” including hearing expressions that hegemonic men and women who discredit OTM men often employ, such as: “In my house, I fuck when I want, and when I want is when my wife says.” This example and the others described in this section unveil some language uses that perpetuate unequal situations with regard to gender and sexual relationships. In fact, instead of being inoffensive verbal jokes, they become relevant barriers that undermine OTM men's self-confidence.

### Transformative Communicative Acts: NAM-Related Self-Confidence

The offensive sexual statements discussed in the previous section can have different effects when self-confidence-based language is used by non-hegemonic men. The interviewees described several examples of this situation and their consequences for the offenders and society as well. These men, considering our analysis showing evidence that responds to the NAM model, experience changing dynamics in their daily life, as Alejandro's story demonstrates. Thus, Alejandro commented on how his involvement with a men's movement, where discussions about NAM attitudes take place, has helped him significantly in developing his self-confident attitude. He highlights the importance of this aspect and shows how displaying this attitude in front of others is an element that contributes to changing heterosexual women's sexual desires. The communicative act explained here perfectly exemplifies this change. Alejandro describes how his best friend, Pedro, generates attention from women when he uses self-confident body language. Despite being sexually scorned by his wife, Lucía, when situations arise that uplift his self-confidence, the responses are radically different. Therefore, Alejandro decided to invite Pedro to his university class on social work because, as a university professor, he has conducted a large amount of investigation into social rights. Alejandro knows that this situation benefitted Pedro, as that day the audience was impressed by Pedro's lecture and he generated a lot of attention.

More than the physical attractiveness is the appeal of self-confidence, with the attitude that you show, because Pedro has also given lectures at the university. He was talking about social rights, and he started to talk in front of the students and professors, and he was very comfortable because it is an issue they understand, and this self-confidence that he showed generated a lot of sexual appeal.

As mentioned before, changing women's specific attraction patterns became possible because of these types of body language and communicative acts; these elements were strongly marked by self-confidence in his sexual appeal. The following comment, made by Alejandro's female university colleague regarding his friend, demonstrates how women who do not often feel sexual desire toward non-hegemonic men can change this desire because of the NAM self-confident attitude: “The Director of social work studies, who is a friend of mine, told me, “Hey, please give me his phone number” jokingly, because at that moment he seemed very self-confident.”

Alejandro's own experience is also illustrative of the power of these transformative reactions, particularly how it is useful to speak confidently about sex and attraction in the face of offensive sexual statements. Alejandro enjoys playing amateur soccer and he used to share locker-room with men who follow the DTM model. For that reason, he commonly has to deal with various situations where certain sexual statements are made by his colleagues. His colleagues' mockery is often connected to his egalitarian attitude that, for them, is totally disconnected from sexual success. Alejandro combats these types of statements by using responses where he clarifies that his sexual life is very active and delightful.

Friends: I am going to fuck and you, Alejandro, are going to wash the dishes and iron!

Alejandro: I do the housework because we distribute the tasks, like when we make love.

Friends: She doesn't let you go out and, on top of that, you don't fuck!

Alejandro: You are very worried about my sexual life—maybe you are the one who doesn't fuck? Because I have never complained about this. I am very satisfied.

The interactions that Alejandro has in his weekly meetings with soccer colleagues demonstrate a reality that scientific literature had already underlined: the reproduction of chauvinist attitudes in men's traditional spaces, such as sports (Anderson, 2011). However, Alejandro's attitude, based on the self-confidence that characterizes NAM men, changes his colleagues' reactions. As he affirmed in the interview, they respect him: “After my answer, they laugh and respect me and leave me alone.”

In the same way, Xavier's observations on communicative acts that address offensive sexual statements are powerfully connected with self-confidence as well. Since he was a child, Xavier has had to deal with offensive situations so as not to follow the DTM model. He made it clear that he wanted egalitarian and passionate relationships, but he found some traditional attitudes in others' reactions. In this regard, some of the situations he described in the daily life narrative refer to him. One situation took place at the university where he met a girl that wished to become intimate with him, but Xavier rejected her because he did not like her arrogant and disdainful attitude toward non-hegemonic men. Consequently, she began to insult him and questioned his sexual orientation, but he answered to her very confidently:

There was once a girl in my class who wanted to hook up with me, and she was very insistent. I did not want to do this, and she said to me in a bad mood: “Are you a homosexual?” And I responded to her: “I hook up with whom I want, when I want.”

This quick reaction has positive consequences because this girl did not make any further offensive statements about Xavier's sexuality. Comparable reaction succeeded with Xavier's current girlfriend when he adopted this attitude, but with sexual situations in this case. If he is self-confident and makes jokes when indifference appears during sex, his girlfriend drastically modifies her attitude and her attraction increases.

If I am self-confident then her eyes start to shine, it is automatic, and if I am not self-confident, she disconnects. If I'm feeling good I say: “You are controlling everything again, right? I notice that you are relaxing” or something like that. She already knows what happens… then I say to her: “You are ordering me around again because you feel like partying. You are already in fifth gear”! Then she laughs, she normally understands it and changes. I feel better about myself, and she is more interested. I feel ok because I see how she changes.

Xavier is extremely aware of the importance of self-confidence and having high self-esteem to keep his girlfriend's sexual desire alive. The relevance is illustrated in the following quote, where he provides an example of the type of language he usually utilizes in such situations. Xavier relates how, during a date where they were intimate, his girlfriend started to yawn. He described how this situation engendered a loss of sexual drive, and to get the situation to how it was previously, he started to make non-offensive jokes that maintained his girlfriend's interest.

In a situation where she is ignoring you or yawning, if you feel self-confident with what you want, you can make a joke to clarify what it is that you want, what you do not want and what interests you. From that moment, you can see how she reacts. You can say: “Well, now you are relaxing, you are starting to yawn.” It is a way to say that I do not want to be with her in this way.

Thus, Xavier feels very relevant and has such a high level of confidence that he does not have to accept any attitude of this type. In that case, when something similar has happened, he has chosen to immediately stop the date several times. This type of reaction has become crucial for maintaining the passion in his relationship: “So my reaction is to stop, and I say, “No problem—if you are feeling like this today, it is not the best day to be together. You can go home” because I do not enjoy being with her in this way.” Xavier describes how his girlfriend alters her sexual desire toward him when these types of communicative acts are performed. Xavier notes that this change is due to his commitment to be self-confident: “When I win Maria's respect and I am coherent with what I am saying and feeling, I see that she is very excited sexually, and I feel comfortable and more self-confident.”

Like Xavier, Teresa clarified in her interview how her husband, Manuel, is self-confident about his sexual appeal. Since they started their relationship, he made clear what type of treatment he would require. Hence, Manuel demanded equality but at the same time passion and desire. Manuel's commitment to this demand is evidenced in different situations. For instance, Teresa explained one situation where she lived with him and his childhood friends. In that situation, his friends make comments ridiculing his sexual life, but he answered very self-confidently showing that he was very much sexually active:

Friend: Surely you don't fuck much. Teresa is always stopping you. Oh my goodness, Manuel, if only they could see you now.

Manuel: The number of times you do it doesn't matter, but the quality does.

Self-confidence of one's sex appeal is one of the elements that Teresa highlights the most when recounting her daily life narrative, which has drastically influenced her sexual desire. Prior to meeting her current partner, she used to lose interest in other men, but now it is different because Manuel's attitude generates desire in her:

He helps me to see the relationships from another perspective, with this attitude of self-confidence, and he says: “This is what I think, and just because I am like that does not mean that I will follow you or do what you want.”

Teresa insisted that this is a question of willingness, based on self-confidence, and the long conversations they both usually had about their relationship increased her attraction toward him:

He always has been clear about how he wanted to live with me, and he has always explained this to me perfectly with very long conversations. I like these conversations very much, and every time we speak, the respect and desire increases.

Enric's reflections on his daily communicative acts in the workplace are in the same line as previous participants. His self-confidence and sexual-affective fulfilling relationship help him face his boss' sexual and intimidating jokes with very self-confident responses. For instance, a recent situation that Enric experienced with his boss, Robert, shows the nature of these responses. Enric explains how Robert went to his desk and, in front of his co-workers, made an insulting joke and tried to undermine his sexual appeal while Enric was talking with his girlfriend on the phone. He said, “Is your girlfriend controlling your life?” Enric did not become quiet, and he continued talking aloud to his girlfriend saying: “Darling (referring to his girlfriend), wait a moment, because Robert is joking with me, and he wants to have a coffee with me as well, but I am not interested in him.” After this situation, Robert did not insult him anymore, and his female co-workers became interested in Enric's sexual appeal.

## Discussion

Hegemonic masculinity and the reproduction of gendered discourses that foster its privileges are widely explored (Connell and Messerschmidt, 2005; Korobov and Thorne, 2006; Brown and Macdonald, 2008; Duncanson, 2015; Yang, 2020). However, it has also been claimed that the concept of hegemonic masculinity can have a transformative dimension (Duncanson, 2015; Yang, 2020). Thus, studies on the discourses of non-hegemonic men, such as the egalitarian men's movement, have been developed (Connell and Messerschmidt, 2005; Connell, 2012; Ramirez et al., 2015). The latter shows evidence of the creation of men's public messages that do not replicate traditional chauvinist mottos; on the contrary, they construct language that prioritizes gender equality. In spite of all these studies, several recent analyses show a gap in this dichotomy (Serradell et al., 2014; Puigvert et al., 2019). Puigvert et al. (2019) demonstrated the existence of an attraction pattern promoted in socialization leading to consider violent men as the most sexually desirable. These findings denote that there is a dominant coercive discourse that fosters the link between violence and desire in the socialization process, and, simultaneously, dissociates egalitarian men and sexual attraction. Serradell and colleagues illustrate how young people do not consider egalitarian messages as attractive, thus creating a double standard (Serradell et al., 2014). At the sexual level, this means that there are men who are exciting—that is, men who are described with language full of desire with whom heterosexual women want to have sex. In contrast, there are “good boys,” “wimps,” very egalitarian and understanding men, who are defined by language that lacks desire and sexual connotations.

However, recent research has also shown that the coercive discourse that forcefully associates violence with attraction can be reversed through appropriate interactions. Racionero-Plaza et al. (2018, 2020) demonstrated that certain interactions have the capacity to raise a critical consciousness about the dominant coercive discourse in sexual-affective relationships and thus the approach to relationships can be transformed. In this vein, NAM's communicative acts also have the capacity to reverse the coercive discourse. By describing the offensive sexual statements made in front of oppressed men, new elements to understand the preceding analyses emerge. Thus, our data sheds new lights on previous analyses of language use with regard to non-hegemonic men (Portell and Pulido, 2012). On the one hand, our findings show how non-hegemonic men face insulting messages that question their manhood and undermine their sexual appeal from heterosexual women who associate desire and sexual excitement with the DTM model (Duque, 2006; Puigvert et al., 2019). These elements reduce their capacity to exist outside of the aforementioned double standards. In contrast, men who follow NAM's premises, when their communicative acts are based on self-confidence in the sexual arena, as well as when attracting women, transform this inequality spiral. NAM's communicative acts can obliterate offensive sexual statements. Thus, they can generate desire and equality toward NAM and contribute to undermine the link between violence and desire, and so erode the attraction toward traditional hegemonic masculinities. NAM men's communicative acts have a central role in this transformation as scientific literature had already indicated (Díez-Palomar et al., 2014). In order for these acts to be transformative, the results show that it is necessary that these men combat the coercive discourse with confident behavior at the sexual level, so that they can foster changes in women's attraction patterns.

These last remarks have several social implications that we believe should be taken into account in further research. Although these social implications have already been noted in research (Duque, 2006; Valls et al., 2008; Aguilar, 2009; Racionero-Plaza et al., 2018; Rios-Gonzalez et al., 2018; Puigvert et al., 2019; Duque et al., 2020), more analyses are needed to understand how NAM men's communicative acts, based on self-confidence in sexual and attraction issues, are shaped in various daily-life contexts such as in schools and in leisure time. In this regard, there are some schools around the world that implement actions that consider the NAM approach (Padrós, 2014; López de Aguileta et al., 2020; Racionero-Plaza et al., 2020).

In short, there are certainly more stories of men, women, boys and girls who change their conceptions of attraction in their language uses. Stories like those of Teresa, Xavier, Enric, and Alejandro show evidence of the consequences of employing specific adjectives or having particular attitudes. Hence, following the premises of CM, researchers have the responsibility of making these stories more visible because they are offering important knowledge to achieve social change in affective and sexual relationships that citizens are requesting.

## Data Availability Statement

The raw data supporting the conclusions of this article will be made available by the authors, without undue reservation.

## Ethics Statement

The studies involving human participants were reviewed and approved by the Community of Research on Excellence for All's (CREA) Ethics Committee with the number 202102231. The participants provided their written informed consent to participate in this study. Written informed consent was obtained from the individual(s) for the publication of any potentially identifiable images or data included in this article.

## Author Contributions

HZ-E and NG-F conceived the idea of the study. NG-F, HZ-E, and MG contributed with the literature review. HZ-E wrote a draft of paper with the support of NG-F and MG. NG-F and MG conducted a review of the draft and provided feedback. HZ-E included the feedback and wrote the final version of the manuscript. All authors have read and agreed to the submitted version of the manuscript.

## Conflict of Interest

The authors declare that the research was conducted in the absence of any commercial or financial relationships that could be construed as a potential conflict of interest.
